# 3D MR neurography targeted peripheral nerve ablation with MR-guided high intensity focused ultrasound (MR-HIFU): initial results of a feasibility study in a swine model

**DOI:** 10.1186/2050-5736-3-S1-P74

**Published:** 2015-06-30

**Authors:** Robert Staruch, Merel Huisman, Michelle Ladouceur-Wodzak, Avneesh Chhabra, Rajiv Chopra

**Affiliations:** 1 Philips Research, Dallas, Texas, United States; 2 University Medical Center Utrecht, Utrecht, Netherlands; 3 University of Texas Southwestern Medical Center, Dallas, Texas, United States

## Background/introduction

MR-guided HIFU is an effective treatment for metastatic bone pain through periosteal nerve ablation,[1] and is being investigated for treating back pain through facet joint denervation.[2] For peripheral neuropathy, ultrasound-guided HIFU has been investigated preclinically as a means of achieving either an irreversible conduction block to treat severe spasticity,[3] or a reversible partial conduction block to alleviate chronic pain.[4] However, ultrasound offers limited visualization of deeply situated pelvic nerves [5] and lacks the ability to measure thermal dose, which predicts the extent of changes in peripheral nerve histology and function.[6] Recently developed diffusion-prepared 3D MR neurography imaging techniques with fat suppression and nerve-selective T2-weighting [7] could improve targeting accuracy over ultrasound guidance. We present initial investigations into the use of MRI to guide HIFU ablation of peripheral nerves in a swine model. The objectives were 1) to evaluate the feasibility of identifying peripheral nerves using MR neurography on the clinical MR-HIFU system, 2) to monitor HIFU ablation of peripheral nerves using MR thermometry, and 3) to evaluate the ability to measure thermal lesions in peripheral nerves using contrast-enhanced T1-weighted images and thermal dose maps calculated from MR thermometry.

## Methods

Experiments were approved by the local Institutional Animal Care and Use Committee. Volumetric MR-HIFU was used to induce seven thermal lesions in the sciatic nerves of three pigs. 3D MR neurography and T1-weighted images at 3T were used for target identification and treatment planning. A single 8 or 12 mm treatment cell was used to cover the full width of each targeted nerve. Ultrasound exposures were performed under MR thermometry guidance in five image planes across the HIFU beam, and one plane along the beam axis. Sonications were performed at 1.2 MHz with acoustic power ranging from 160 to 300 W over fixed durations of 20 or 36 seconds (energy 3.2 to 10.8 kJ). Ablation dimensions were measured and compared using thermal dose maps, contrast-enhanced T1-weighted images, and gross pathology.

## Results and conclusions

**Figure 1:**
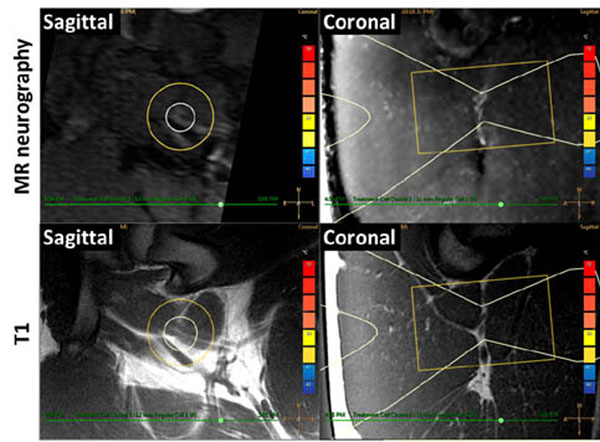
MRI targeting of peripheral nerve. Pig sciatic nerve is hyperintense on oblique 3D MR neurography (top), and isointense on T1 where dual-bundle fascicular structures are seen against surrounding fat (bottom). HIFU beam overlay (white) indicates target.

**Figure 2:**
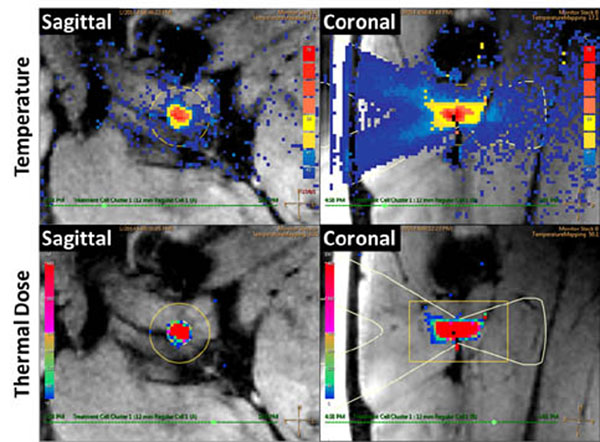
MR thermometry guidance of HIFU thermal ablation of peripheral nerves. Temperature maps (top) indicate peak temperature of 82.5°C. Thermal dose maps (bottom) predict lesion size of 12.2 x 29.2 mm for a 12 mm diameter treatment cell.

**Figure 3:**
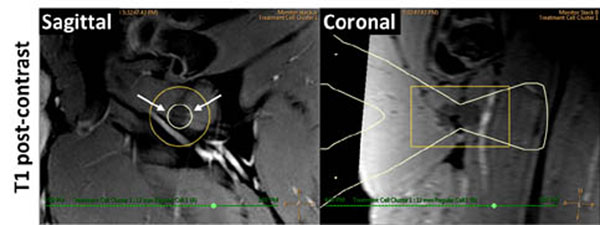
Evaluation of thermal lesions in peripheral nerve. Non-enhancing region visible on post- contrast T1 images. HIFU beam path overlay (white) indicates target location.

**Figure 4:**
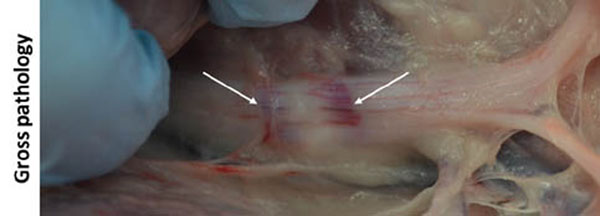
Photograph of HIFU-induced thermal lesion in pig sciatic nerve. Coagulated region appears blanched with a hyperemic rim (arrows).

All targeted sciatic nerves were identifiable on MR neurography and T1-weighted images (Fig 1). For sonications at 160 to 300 W, MR thermometry measured peak temperatures of 60.3 to 85.7°C, with 240 equivalent minute thermal dose diameters of 8.5 to 15.9 mm (Fig 2). Thermal lesions were visible on late phase contrast-enhanced T1 (Fig 3), with dimensions matching the coagulated region observed at necropsy (Fig 4). Our preliminary results indicate that targeted peripheral nerve ablation is feasible with MR-HIFU. Diffusion-prep 3D MR neurography has potential for guiding therapy procedures where either nerve targeting or avoidance is desired.

## References

[B1] Hurwitz Magnetic Resonance-Guided Focused Ultrasound for Patients With Painful Bone Metastases: Phase III Trial Results JNCI 2014 106 5 10.1093/jnci/dju082 PMC4112926 24760791

[B2] Weeks MRI-guided focused ultrasound (MRgFUS) to treat facet joint osteoarthritis low back pain-case series of an innovative new technique Eur Radiol 2012 22 2822 35 10.1007/s00330-012-2628-6 22935902

[B3] Foley Image-guided high-intensity focused ultrasound for conduction block of peripheral nerves Ann Biomed Eng 2007 35 109 19 17072498 10.1007/s10439-006-9162-0

[B4] Foley Effects of high-intensity focused ultrasound on nerve conduction Muscle Nerve 2008 37 241 50 10.1002/mus.20932 18041054

[B5] Fritz Magnetic resonance neurography-guided nerve blocks for the diagnosis and treatment of chronic pelvic pain syndrome Neuroimag Clin N Am 2014 24 1 211 34 10.1016/j.nic.2013.03.028 24210321

[B6] Vujaskovic Effects of intraoperative hyperthermia on peripheral nerves: neurological and electrophysiological studies Int J Hyperthermia 1994 10 1 41 9 10.3109/02656739409009330 8144987

[B7] Yoneyama Rapid high resolution MR neurography with a diffusion-weighted pre-pulse Magn Reson Med Sci 2013 12 2 111 9 10.2463/mrms.2012-0063 23666153

